# A Letter from Philadelphia

**Published:** 1885-10

**Authors:** Harry Huzza

**Affiliations:** Philadelphia


### A LETTER FROM PHILADELPHIA.

Opening of the medical colleges—Jefferson's new professor of chemistry—workings of the new anatomical act, etc.

---

*Editors Atlanta Medical and Surgical Journal:*

I write to-day according to promise. There has been nothing of special interest to the profession as yet, however it may have been to me. The preliminary lectures began Thursday, September 17th, with a lecture by Professor Forbes, on the "Anatomy of the Eye." He was followed by Professor Holland, the new professor of chemistry, on "Magnetism." The students are so far delighted with him, and he bids fair to be a favorite.

Over two hundred and fifty students have so far matriculated at Jefferson. This is of course no certain indication of the attendance this year, but at the same time it shows there will be a arge number, very likely above the average. The method of distributing seats, in the order of matriculation, brings the second year students back early.

At the University of Pennsylvania prospects are equally bright for a good attendance. Their buildings make a fine show, with all their advantages of plenteous space, grassy parks, and within all the latest scientific apparatus and modern improvements.

The anatomical act is working splendidly, and the supply of material for dissection is ample. This will greatly facilitate the workings of the medical colleges here.

At the clinics there has been nothing so far of special interest. A case of spindle-celled sarcoma of the thigh in a girl nine years was presented as showing the persistence of this disease. There had been two previous operations, in which the tumor was removed and the bone scraped, but they were fruitless. Amputation became necessary, and was performed before the class by Dr. Hearn.

In the Medical Clinic were presented several cases of aphasia, which attracted my attention from the article in THE JOURNAL. One was the case of a telegraph worker, engaged in stringing wires. While on a high cross-bar of a telegraph pole, he was struck in the eye by a roll of wire thrown to him; fell and caught by arm and shoulder on a lower bar. Remembers nothing after being struck, except a rope being put around his body to lower him.

These cases were attributed to an injury of the blood vessels of the third frontal convolution, the speech-center of Broca. The second case was specific in origin. Patient fell frequently; forgot wods, but had no paralysis. Diagnosed as miliary aneurism of small branches of middle part of the meningeal artery, the occasional rupture of these small aneurisms producing the spells of insensibility.

The colleges are opening up well. The students (especially the new ones) are getting to work, and the wheels are being "greased," preparatory to the regular six months' journey.

Very truly, HARRY HUZZA.

PHILADELPHIA, September 18, 1885.

